# Surgical Cases Treated in Buffalo General Hospital during Service Term of Dr. J. F. Miner

**Published:** 1871-01

**Authors:** D. W. Harrington

**Affiliations:** Acting Resident Physician


### ART. VIII.—*Surgical Cases treated in Buffalo General Hospital during Service Term of* DR. J. F. MINER.

REPORTED BY D. W. HARRINGTON, ACTING RESIDENT PHYSICIAN.

David Hickey, aged 51; occupation, sailor; admitted into hospital Nov. 2d; suffering from an injury received two weeks before.

Whilst steering during a storm, the deck being slippery, he had fallen, and the wheel coming round with force, the spokes or handles struck him across the lower portion of the back and hips; but there was no bruise when admitted into hospital. In walking, he presented an appearance similar to that seen in progressive locomotor ataxy. His pulse was feeble and intermittent; he complain ed of pain in back and head; his hands trembled so that he had no control over them, and had to be fed.

This condition had been coming on gradually since the injury. He had been in this hospital during the month of July, from a slight injury to hand. He was then patient, kind and agreeable; now he is fretful, peevish and irritable. He continued to grow worse until about Nov. 15th, when he became delirious and stupid. He soon became semi-comatose, in which condition he remained until his death, Dec. 1st.

He could be aroused, and made to take food and stimulants; would answer questions in a muttering, disconnected manner. He had control over his evacuations, and would have them when urged to do so. *Post mortem* not allowed.

*George Getsinger*, admitted into Hospital June 1st—a private patient of Dr. Miner's—with fracture of the fibula, and separation of the tibia at the lower epiphysis, the two bones having been driven through the soft parts, upon the inner side of the leg, denuding them of periosteum, lacerating and dividing the muscles and integument, in the most fearful manner. The fracture and displacement had been adjusted, and leg dressed, by Dr. Miner, before admission. After two weeks, the roughened portion of the bones, which had been driven through the soft parts, clothing, boot, &c., and thus denuded of periosteum, had manifested no indications of returning life; and, June 11th, exsection of both bones was made; two inches in length, being removed from the upper fragments.

Extension was immediately applied, and passive motion as soon as practicable, He was discharged from hospital Sept. 7th. He is now using neither crutch or cane; has perfect motion in the ankle; and, what is quite remarkable, there is shortening of only one inch, the other inch supplied by deposit of new bone.

This is a great achievement in what is called conservative surgery; and indicates, as strongly as any one case can, that, as a general principle, amputation should not be made on account of injury to bone, while the vascular supply is not seriously interfered with. This injury was such as was formerly treated by amputation, and probably many surgeons would not even now attempt to save such a leg, and yet the result is as good as is generally obtained in simple oblique fracture.

